# Prospects and Challenges for T Cell-Based Therapies of HCC

**DOI:** 10.3390/cells10071651

**Published:** 2021-06-30

**Authors:** Norman Woller, Sophie Anna Engelskircher, Thomas Wirth, Heiner Wedemeyer

**Affiliations:** Clinic for Gastroenterology, Hepatology and Endocrinology, Hannover Medical School, 30625 Hannover, Germany; engelskircher.sophie@mh-hannover.de (S.A.E.); wirth.thomas@mh-hannover.de (T.W.); wedemeyer.heiner@mh-hannover.de (H.W.)

**Keywords:** immune checkpoint inhibition, hepatocellular carcinoma, tumor surveillance, immunotherapy, T cell responses, treatment failure, CAR therapy, chronic hepatitis, HBV, HCV

## Abstract

The scope of therapeutic options for the treatment of hepatocellular carcinoma (HCC) has recently been expanded by immunotherapeutic regimens. T cell-based therapies, especially in combination with other treatments have achieved far better outcomes compared to conventional treatments alone. However, there is an emerging body of evidence that eliciting T cell responses in immunotherapeutic approaches is insufficient for favorable outcomes. Immune responses in HCC are frequently attenuated in the tumor microenvironment (TME) or may even support tumor progress. Hence, therapies with immune checkpoint inhibitors or adoptive cell therapies appear to necessitate additional modification of the TME to unlock their full potential. In this review, we focus on immunotherapeutic strategies, underlying molecular mechanisms of CD8 T cell immunity, and causes of treatment failure in HCC of viral and non-viral origin. Furthermore, we provide an overview of TME features in underlying etiologies of HCC patients that mediate therapy resistance to checkpoint inhibition and discuss strategies from the literature concerning current approaches to these challenges.

## 1. Introduction

Patients with advanced stages of HCC face a poor prognosis. Liver cancer is the third leading cause of cancer-related mortality worldwide [1]. It is still a difficult-to-treat disease, despite several treatment options, such as liver transplantation, systemic treatment with chemotherapy, loco-regional treatment, such as transarterial chemoembolization (TACE) and radioembolization or treatment with sorafenib, lenvatinib, or other multi-kinase inhibitors [2,3,4]. Untreated HCC has a 5-year overall survival rate of less than 10% and curative options in advanced stages, when the disease is usually detected, are rare [5]. Recently, prognosis of HCC had improved remarkably with the implementation of immune checkpoint inhibition (CPI) into the treatment schemes as we will discuss in detail. The agents that are commonly used for CPI are antibodies inhibiting the CTLA4 pathway, such as ipilimumab, and the PD-1/PD-L1 pathway, such as pembrolizumab and atezolizumab. CTLA-4 is a homologue of CD28 that binds to members of the B7 family during T cell activation by antigen-presenting cells and has a higher affinity than does CD28. The interaction of PD-1 with PD-L1 keeps T cells from killing tumor cells, whereas blocking this interaction can allow for cytotoxic responses to lyse tumor cells.

Not even a decade has passed from the time when concrete evidence was found that lymphocytes can prevent tumor development [6] until the first clinical studies of checkpoint inhibition confirmed increased survival in patients [7,8]. In 2010, patients with metastatic melanoma benefitted from treatment with ipilimumab, an inhibitor of the CTLA-4 pathway. PD-1/PD-L1 checkpoint inhibitors soon followed suit, also showing effectiveness of checkpoint inhibition in other tumor entities [9,10]. It was realized that CPI elicits T cells against cancer neoantigens as the main drivers of responses [11,12]. Since occurrence of positive clinical studies, a plethora of checkpoint inhibitors targeting PD-1 and PD-L1 has been approved and tested in clinical trials in a great variety of cancers. The results of phase 1/2 and 2 clinical trials in HCC patients with single use of blocking antibodies of the PD-1 pathway led to approval of these agents by the United States Food and Drug Administration (FDA) for the treatment of HCC [13,14]. However, the first phase III trials failed to reach the predefined endpoint both for nivolumab as a first line therapy and pembrolizumab (both inhibitors of the PD-1 pathway) as second line treatment [15,16]. Still, both trials confirmed an overall response rates (ORR) of 15–20% observed in the phase II trials. However, among these responding patients, complete responses defined as disappearance of vital tumors were almost non-existent.

These clinical results show that efficacy of CPI treatment of HCC lagged behind other tumor entities, primarily metastatic melanoma (ORR 61% [17]) and Hodgkin lymphoma (ORR 87% [18]), among others [19,20]. This was true until recently the clinical study IMbrave150 was published that combined CPI (atezolizumab, a PD-L1 checkpoint inhibitor) with inhibition of angiogenesis (bevacizumab, targeting vascular endothelial growth factor (VEGF)). Overall survival rates at 12 months were significantly higher in the atezulizumab + bevacizumab arm (67.2%; 95% CI: 61.3–73.1) compared with the sorafenib arm (54.6%; 95% CI: 45.2–64.0). It reported a hazard ratio for overall survival of 0.58 in favour of the combination therapy. This represents a 42% reduction in the risk of death compared with the previous first-line treatment with a tyrosine kinase inhibitor (TKI) sorafenib [21]. Additionally, the study also provided HCC patients a perspective for complete responses, as was the case in 5.5% of patients in the combined treatment group (18/326, according to independent review facility-assessed Response Evaluation Criteria in Solid Tumors (RECIST) v1.1). These clinical results are unprecedented with regard to the treatment outcome, demonstrating the potential of CPI within combinatorial treatment regimens [21]. The IMbrave150 study changed therapy guidelines for first line therapy from tyrosine kinase inhibition to immunotherapy [22] and therefore it is worthwhile to take a closer look at potential underlying mechanisms of CPI that facilitate immune-mediated clearance of HCC cells. Moreover, the key question that has to be addressed is how the immune system can be thoroughly stimulated by pharmacological intervention for a long-term effective treatment, as tumor immunity appears to be crucial in regimens of HCC. In addition, this knowledge could be used for other T cell-based therapies, including therapeutic vaccination and infusion of ex vivo expanded T cells. 

## 2. The Vade Mecum of HCC Treatment Is Now Based on Pillars of Cellular Immunity by Combining Checkpoint Inhibition with Anti-Angiogenesis Treatment

The best objective response rates (ORR), derived from clinical studies of HCC, all include checkpoint inhibitors. A phase Ib study of the multi kinase inhibitor lenvatinib targeting VEGFR1, VEGFR2, and VEGFR3 plus pembrolizumab has an ORR of 36% [23], PD-1 inhibition with nivolumab and ipilimumab shows an ORR of 32% [24] followed by atezulizumab and bevacizumab treatment with 27.3% ORR [21]. Relating to long-term efficacy and safety profiles, the latter regimen is the first-line treatment of choice.

Angiogenic factors such as hypoxia-induced and tumor-microenvironment (TME)-derived VEGF is capable of downregulating adhesion molecules on endothelial cells. Expression of intercellular adhesion molecule 1 (ICAM-1) and 2, and vascular cell adhesion molecule 1 (VCAM-1) inhibit T cell adhesion [25,26]. Consequently, elevated levels of VEGF in the TME have been correlated with immune exclusion of T cells within the tumor tissue [27]. Regulatory T cells (Tregs), myeloid-derived suppressor cells (MDSCs), and tumor-associated macrophages (TAMs) are recruited to HCC tumors, mediated by VEGF and hypoxia-inducible factor 1 (HIF-1). This links tumor tolerance by failure of T cell immune surveillance to hypoxia [28,29]. Thus, it is a strong rationale to combine VEGF inhibition with CPI, as investigated in the phase III IMbrave150 trial. Therapy with atezolizumab blocks PD-L1 expressed on immune cells and tumor cells. Blocking PD-L1 prohibits interactions with the ligands PD-L1 and CD80. PD-1 is a checkpoint inhibitory receptor that is expressed on antigen-primed T cells in infection and cancer [30]. This receptor regulates T cell proliferation and tolerance and is involved in tumor evasion and T cell exhaustion [31].

**Figure 1:**
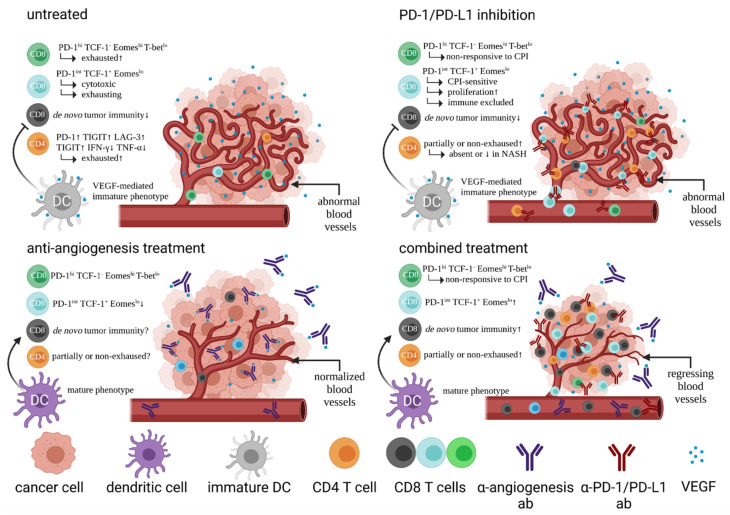
Effects of PD-1 or PD-L1 inhibition, anti-angiogenesis treatment, and combined treatment on immune cells and tumor tissue in HCC (abbreviations: ↑/↓ high/low; marker^hi/lo^ high/low marker expression).

This effect is reversible by inhibiting VEGF:VEGF receptor-2 interactions [32]. HCC tissue is highly neovascularized and accessible for inhibition of angiogenesis, as these tumors also show a high microvessel density [33]. Regarding the combination of atezolizumab and bevacizumab, there is evidence that combined treatment enhances antigen-specific T cell migration, potentially through vascular normalization and endothelial cell activation. The combined regimen leads to increased counts of CD8 T cells, increased Th1- and T-effector markers, intra-tumoral major histocompatibility complex (MHC)-I expression and chemokine levels [34]. This demonstrates that bevacizumab has more effects than merely inhibition of angiogenesis, with a particular benefit for the immune system. Apart from structural remodelling of the tumor blood vessel bed by anti-VEGF therapy that facilitates T cell infiltration, there is also data that it reverses the inhibitory effects of VEGF on dendritic cell (DC) maturation that leads to reduced T cell priming [35]. As single-agent activity in HCC, response rates of atezolizumab and bevacizumab are quite similar. With 17% for atezolizumab in a Phase Ib study and 14% for bevacizumab in Phase II studies, it raises the question whether the effects are additive or complementary due to the different modes of action [36,37,38,39]. Both compounds mediate their effects on different parts of the immune system that are considered complementary and thus are more likely to elicit tumor immunity and increasing response rates. On the one hand, the IMbrave150 shows a response rate of 27%, which would suggest an additive effect. On the other hand, a majority of patients (88%) maintain their response long term, i.e., for six months or longer. Figure 1 shows the proposed mechanism of combined regimens using CPI and inhibition of angiogenesis.

## 3. Biomarkers and Immunological Classification of HCC

Predicting therapeutic benefit prior to or shortly after therapy starts by biomarkers is a well sought-after aim in clinical oncology [40]. So far, the most common marker that is correlated to a therapeutic response in other tumor entities is the expression of PD-L1 in tumor-tissue and the tumor mutational burden (TMB). However, these parameters have not been shown to reliably predict treatment responses in HCC patients receiving checkpoint inhibitors [41,42,43]. One study even questions the dominant role of neoantigens in HCC for CPI due to the relatively low mutational burden compared to malignant melanoma [44]. The lack of common markers for the prediction of the treatment response of HCC led to other assessments as we will see later.

Anti-tumor immunity can appear concomitantly with tumor progression. This observation is called the “Hellström paradox” according to a study from 1968 that found humoral and cellular components with tumoricidal activity in patients with growing tumors [45]. These findings suggests that activity of tumor-directed immunity must outpace tumor cell proliferation to reach a threshold of net reduction in the overall tumor mass. This view conforms also with the state of equilibrium derived from the hypothesis of immunoediting [46]. Accordingly, cancer immunotherapy aims at amplification of existing tumor immunity or de novo generation in order to tip the scale towards favourable outcomes [47]. With regard to HCC, the stratification of etiologies for the clinical outcome may help to dissect and understand effects of signaling pathways and immune cell phenotypes on immunotherapy responses [48]. It is important to distinguish the non-responsiveness to cancer immunotherapy between the failure of triggering an immune response and the functional failure of the elicited response. Here, primary, adaptive, and acquired resistance can be differentiated [49]. Acquired resistance is an important, but often underestimated, clinical parameter showing that responses are mostly temporary. Thus, the design of future clinical studies should include strategies to maintain already existing immune responses. The overall designation of all immune factors within a host that eventually leads to the killing of cancer cells is briefly called the cancer immune cycle [50]. Any interruption within that sequence or the functionality of essential networks of the cycle leads to a complete abortion and eventual failure of tumor rejection. It is a convenient tenet and the ultimate reason to explain resistance to cancer immunotherapy and treatment failure of CPI. A more refined view on the clinical effects of checkpoint inhibitors is the cancer immune set point. This views immunity to cancer as a complex set of tumor, host and environmental factors. These factors govern the magnitude and timing of the anticancer response [51]. Consistent with the observation that HCC develops in a complex environment of chronic hepatitis and fibrosis, likewise the genomic landscape has been described as highly complex and heterogeneous [52,53]. More suitable for predictions appear multi-omics approaches that have been proposed for immune profiling of HCC. A study of Sia et al. analysed 956 HCC samples and found that about 25% of HCC have markers of an inflammatory response with high expression levels of PD-1 and PD-L1, cytotoxic marker expression, such as an interferon gamma (IFN-γ) signature, and low levels of chromosomal aberrations. The immune class correlated with better overall survival [54]. Additionally, the subgroup of this cohort was either characterized by an adaptive T cell response or an exhausted immune response that allowed stratification of an active and exhausted immune subclass. The active immune subclass showed signs of an ongoing cytotoxic response, in which IFN-γ and granzyme B signatures are present. In contrast, the immune-excluded subclass was dominated by signature of T cell exhaustion, suppressive myeloid cells, and tumor growth factor-β (TGF-β). In another study, Zhang et al. performed immune profiling of HCC and defined three groups that suggest differentiation into immunocompetent, immunosuppressive, and immunodeficient subtypes [55]. Expression level analysis of CD45 and Foxp3 in immunohistochemistry (IHC) allowed for correlative classification of the treatment outcome in this study. The immunocompetent subtype was CD45^hi^ and FOXP3^lo^ showing infiltration of αβ and γδ T cells. Furthermore, HCCs of the immunosuppressive subtype stained CD45^hi^ and FOXP3^hi^ indicating regulatory T and B cells, as well as tolerogenic macrophages and immunosuppressive molecules, such as PD-1/PD-L1, TGF-β, VEGF, T cell immunoglobulin and mucin domain containing protein 3 (TIM-3) and interleukin-10 (IL-10). The immunodeserted subtype showed a CD45^lo^ phenotype with a significant reduction of immune cell infiltration [55]. A similar classification of the immune composition of HCC by a study investigating 158 HCC patients was proposed to distinguish three immune-subtypes: Immune-high, immune-mid, and immune-low. Increased plasma/B cell and T cell infiltration in the immune-high subtype were identified as independent positive prognostic factors [56]. These promising studies show that an in-depth immune profiling potentially combined with genetic approaches may lead to stratification of HCC for appropriate prediction of the outcome. In addition, these studies suggest that T cells with distinct properties exists that prevent tumor outgrowth.

## 4. The Immune Landscape of HCC

An immune landscape of cancer refers to a complex network of the immune cell composition of the TME including cytokines and cell ratio patterns, as well as genetic features of tumor tissue. The latter includes intratumoral heterogeneity, aneuploidy, mutational burden, and expression of immunomodulatory genes that have an impact on the leukocyte levels. Mutations in *CTNNB1*, *Nras*, and *IDH1* are associated with low levels of leukocytes, whereas *BRAF*, *TP53*, and *CASP8* are correlated with high levels of leukocytes. All these factors ultimately contribute to the prognosis [57]. The immune landscape of HCC has recently been investigated by several studies [58,59,60,61]. The main driver of HCC development is cirrhosis of the liver and it has been shown that tumors are inflammation-associated and generate a tumor microenvironment (TME) that is highly immunosuppressive [62]. The immune cell composition underlies fundamental differences according to healthy liver tissue, adjacent tissue, and tumor tissue. Here, total B and T cells are significantly upregulated in tumor tissue, whereas CD8 T cells are abundant in adjacent tissue and tumors. Interestingly, this study also found that the magnitude of Treg cells is significantly higher in adjacent tissue than in tumor tissue [60]. Generally, the presence of Tregs in HCC patients correlates with a poor prognosis [63], whereas tumor infiltrating CD8 T cells are associated with an improved outcome [54,64]. Single CD45^+^ immune cell analysis of the landscape and dynamics in HCC identified lysosomal associated membrane protein 3 positive (*LAMP3*^+^) dendritic cells that did not correspond to any classical DC subset in vivo. These cells can migrate from tumors to hepatic lymph nodes and have the potential to regulate lymphocytes in situ. Moreover, in this study Zhang et al. performed RNA velocity analysis that indicated a directional flow from proliferative to exhausted CD8 T cells [61]. Exhaustion of CD8 T cells is a central issue for the maintenance of immune responses in chronic infections and cancer. With regard to cancer, T cell exhaustion is often associated by interplay with the TME [65]. Exhausted CD8 T cells (Tex) arise as a distinct cell lineage in mice and man and are characterized by high expression of inhibitory receptors, such as PD-1, T cell immunoreceptor with Ig and ITIM domains (TIGIT), TIM-3, and lymphocyte-activation gene 3 (LAG-3). Tex progressively lose effector function and possess a poor memory recall [66,67]. Interference with regulatory checkpoint targets can potentially reverse exhaustion by targeting single or multiple receptors in patients with exhausted HCV-specific CD8 T cells [68], which is also most likely applicable to Tex of cancer patients. A recent study in rodents concluded that TIGIT is the most reliable marker to detect and reverse exhausted Tex in liver cancer [69] and also other studies show the importance of TIGIT as a target for immunotherapy in HCC patients (reviewed in [70]). Hence, regulatory and inhibitory receptors on Tex cells are important clinical targets of immunotherapies to inhibit or reverse Tex progression [65]. The HMG-box transcription factor TOX is a central regulator of Tex. For development of effector (Teff) and memory (Tmem) CD8 T cells TOX is mostly dispensable. However, when it comes to exhaustion, without TOX Tex do not form. Conversely, deletion of *Tox* in tumor-specific T cells residing within the tumor abrogated the exhaustion program. Expression of *TOX* drives Tex commitment by a transcriptional and epigenetic developmental program [71,72]. The gradual process of exhaustion can be further distinguished by additional markers. Early exhaustion is marked by expression of PD-1^int^, TCF-1^+^, and Eomes^lo^. Further chronic antigen stimulation is then thought to lead to terminal exhaustion that is characterized by PD-1^hi^, Tbet^lo^, Eomes^hi^, and a loss of TCF-1 [66,73,74]. In contrast to TCF-1^−^ PD-1^+^ CD8 T cells, TCF-1^+^ PD-1^+^ CD8 T cells have been found to exhibit a proliferative response to CPI and the ability to differentiate into cell lineages of early and terminal exhaustion. Exhaustion of CD4 T cells has also been investigated in preclinical models [75,76], albeit to a lesser extent than CD8 T cells. The exhaustion of CD4 T cells leads to an upregulation of several co-inhibitory receptors, such as PD-1, TIM-3, LAG-3, and TIGIT and is accompanied by reduced pro-inflammatory effector cytokine secretion. A study investigating exhaustion of CD4 and CD8 T cells in human HCC specimen in a single cell approach found that both subsets have distinct profiles when it comes to checkpoint inhibitor molecules, but the study also identified similar features between CD4 Tex and CD8 Tex cells in several other pathways [77]. The process of exhaustion in HCC is attributable to the immunosuppressive microenvironment of the tumor tissue. It has been described that single cell suspensions of freshly collected specimen of HCC tumors showed CD4 and CD8 T effector cells that failed to adequately populate tumor tissue, whereas those cells present exhibited a higher degree of activation compared to their circulating counterparts and occurred with a more exhausted phenotype [78].

The most prominent T cell sublineages that have been described for conveying cellular immunity in cancer are cytotoxic CD8 T cells, CD4 T helper cells, and Treg cells [58]. There is, however, emerging evidence that suggests a more refined view on the T cell landscape to describe all types of T cells that is involved in complex networks of interactions with other somatic compartments such as the TME and adjacent tissues. A seminal study addressing the T cell composition of HCC in detail isolated T cells from peripheral blood mononuclear cells (PBMCs), tumor, and adjacent normal tissues in HCC patients and found that these cells can be divided into subsets based on their molecular and functional characteristics upon single-cell sequencing [79]. Here, Zheng et al. performed deep single-cell RNA sequencing on over 5000 T cells and found that in the CD8 T cell population five consensus clusters emerged. The cluster expressing “naïve” marker genes such as *LEF1* and *CCR7* is found foremost in peripheral blood. Another cluster of CD8 T cells were *CX3CR1*, *FCGR3A*, and *FGFBP2*, commonly found in effector T cells. *SLC4A10* mostly characterized mucosal-associated invariant T (MAIT) cells prevalent in non-tumor adjacent liver tissue. Although MAIT cells recognize bacterial B vitamins such as riboflavin derivatives presented on MR1 [80], it is astonishing that MAIT cell fractions are significantly reduced in HCC tumors compared with adjacent normal tissues and that lower *SLC4A10* expression in HCC correlates with poor prognosis. MAIT cells play an important role as first line of defence in the liver. However, their role in cancers still remain unclear [81]. Interestingly, the study identified two similar CD8 T cell clusters within tumor-tissue. One with high levels of exhaustion markers *CTLA4*, *PDCD1*, and *HAVCR2*, representing Tex and another cluster with shared characteristics to the latter one, but with a *GZMK* signature indicating cytotoxicity that was absent in those exhausted cells. How the tumor-derived CD8 T cell clusters with exhaustion marker genes are intertwined with *TOX*-driven subsets of early and terminally exhausted CD8 T cells remain to be determined [79]. Table 1 provides a brief overview of T cell phenotypes in HCC that are discussed in these studies. Lastly, adenosinergic signaling is an important immuno-metabolic checkpoint in tumors, comprising HCC. Adenosine is frequently being co-opted by tumors to promote growth and impair immunity. Despite a complex regulation of extracellular adenosine, pre-clinical studies have demonstrated significant anti-tumor activity of several agents counteracting the adenosine axis [82,83]. Interestingly, there is encouraging data that coffee consumption interferes with adenosine signaling, is supposed to have beneficial effects on the liver, can prevent liver cirrhosis, and ultimately protect the host from HCC [84,85,86].

## 5. HCC Immune Surveillance by T Cells

The immune surveillance of the liver is a well-studied topic that has revealed several mechanisms throughout different stages of liver cancer development for protection of the host (reviewed in [87]). During the pre-malignant phase of tumor development it has been shown that senescence surveillance is the driving force for the elimination of pre-cancerous and senescent hepatocytes with a secretory phenotype by CD4 T cells and macrophages [88]. Upon progression to the malignant phase, nascent tumor cells are primarily under control of CD4 and CD8 T cells [46]. T cell responses directed against tumor-associated antigens (TAA) in HCC patients are frequently observed and the presence of responses are correlated with survival [89,90,91]. Strong T cell responses directed against TAA are also correlated with suppression of recurring HCC after therapeutic regimens [92]. Well described TAA-responses are directed against alpha-fetoprotein (AFP), human telomerase reverse transcriptase (hTERT), glypican-3 (GPC3), melanoma-associated gene-A (MAGE-A), squamous cell carcinoma antigen recognized by T cells (SART), and New York-esophageal squamous cell carcinoma-1 (NY-ESO-1) [93,94,95,96,97,98].

The tumor mutational burden is, as mentioned above, considered as one important factor for CPI. Mutated neoantigens derive from individual somatic tumor mutations that are bound and presented on human leukocyte antigen (HLA) molecules and are regarded as ideal targets for T cells [99]. HCC has a low to intermediate mutational burden of about 2–8 mutations per megabase, depending on the study [43,44,100,101]. In the study of Ang et al. that analysed 755 patients, only a minority of patients had a TMB-high status (0.8%) and microsatellite instability (MSI-high) barely existed in HCC (0.2%). The occurrence of DNA polymerase alterations (POLE/D) were with 4% more frequent. However, the mutational burden has been found not to correlate with CPI responses [102]. Interestingly, neither TMB nor occurrence of neoantigens was associated with the suggested immune class that predicts favourable responses to CPI [56]. Thus, the exact mechanisms involved in HCC patient responses to CPI remain for the most part unclear. Having said that, there are causes that accurately predict treatment failure of HCC patients, which at least solves a part of the problem. First, activation of the WNT/β-catenin pathway correlates with immune exclusion across human cancers, including HCC [103]. The WNT/β-catenin pathway has been mechanistically investigated in a *MYC; p53*^−/−^ HCC mouse model [104]. The study demonstrated that β-catenin signaling (*CTNNB1*) mediated immune escape of tumors by preventing recruitment of CD103^+^ DCs leading to an early failure of the cancer immune cycle that inhibited generation of robust tumor-specific T cell activity. This effect was rescued by expression of CCL5 by *CTNNB1*^+^ tumor cells. Most importantly, the study found that β-catenin-driven tumors were resistant to PD-1 checkpoint inhibition. Hence, the genetic setup of tumors can inherently influence the immune landscape of HCC that affects therapeutic outcomes.

The importance of HCC immune surveillance by T cells using TAAs has been investigated in patients with liver cirrhosis upon HCV clearance by antiviral therapies [105]. Cirrhotic patients had an increased frequency of CD4 and CD8 T cells that secreted IFN-γ after stimulation with GPC3 peptide pools. Moreover, those patients who developed HCC after antiviral therapy had CD4 and CD8 T cells with significantly lower cytokine release and proliferative capacity compared to those patients that remained tumor-free. Higher magnitudes of GPC3 reactive T cells also delayed diagnosis of HCC developers according to the time of HCC emergence after initiation of antiviral therapy. This study clearly shows the link between the importance of tumor-specific T cells not only in relation to delayed HCC onset, but also for the relevance of immune surveillance for preventing liver cancer [105]. The crucial role of T cells for anti-tumor surveillance has also been demonstrated in a mouse model of liver cancer. Liver tumors were established by transposon-mediated gene transfer. Transposons coding for oncogenic ras linked to potent CD4 and CD8 T cell epitopes was used to transform hepatocytes into nascent tumors with tailored tumor immunogenicity. Potent T cell responses and tumor growth suppression was detected when both, CD4 and CD8 T cell epitopes were expressed. A lack of CD4 tumor-specific epitopes led to induction of robust amounts of tumor-specific CD8 T cells that were incapable of tumor surveillance. On the other hand, presence of CD4 tumor-specific epitopes combined with a lack of CD8 tumor-specific epitopes neither led to CD4, nor to CD8 T cell responses, showing the mutual dependence that is necessary for efficient liver cancer immune surveillance [106].

Although HCC immune surveillance can be regarded as a pivotal mechanism in terms of tumor development, progression, and prognosis, a recent seminal study demonstrated its limitations in non-alcoholic steatohepatitis (NASH), which is an important driver of HCC. The authors observed in preclinical models of NASH-induced HCC that CPI treatment expanded activated PD-1^+^ CD8 T cells but did not lead to tumor remission. Single cell sequencing of cells expressing T cell receptor β (TCRβ) showed gene expression profiles of cytotoxicity and effector-functions together with elevated traits of exhaustion, i.e., *Pdcd1* and *Tox*. PD-1^+^ CD8 T cells accumulated to high numbers of NASH-HCC mice in the liver with a resident-like T cell character. At a first glance, it may appear counterintuitive that accumulation of CD8 T cells within tumor-tissue, that is usually associated with a good prognosis, leads to a failure of immunotherapies in NASH-HCC. However, depletion of CD8 T cells in this model with a preventive setup provided a significant protection from liver damage and HCC development, suggesting that liver CD8 T cells actively promote HCC in NASH. Moreover, the study found similar results in patients. PD-1^+^ CD8 T cells with a residency phenotype were found in two independent NASH cohorts. Interestingly, the magnitude of hepatic PD-1^+^ CD8 T cells directly correlated with body-mass index and the extent of liver damage. Single cell RNA-seq revealed similar gene expression signatures that were also found in mice, i.e., *PDCD1*, *GZMB*, *TOX*, *CXCR6*, *RGS1*, and *SELL*. Furthermore, a meta-analysis of three large randomized controlled phase III trials of immunotherapies in patients with advanced HCC, namely Checkmate-495, IMbrave150, and Keynote-240 [15,16,21], showed that anti-PD-1 or anti-PD-L1 treatment in the control arm led to superior outcome in patients with HBV- and HCV-related HCC, but not in patients with non-viral HCC. However, this meta-analysis did not differentiate between different lines of treatment and between alcoholic liver disease and non-alcoholic fatty liver disease (NAFLD) or NASH. Further investigation revealed that NAFLD was independently associated with shortened survival of patients with HCC after CPI. Hence, this study provides a rationale for stratification of HCC patients according to their etiology of cancer [107]. In line with these results, Heinrich et al. studied the effect of immunotherapy on tumors in the liver in the context of steatohepatitis. Here, application of M30-RNA vaccine or an anti-OX40 antibody led to growth inhibition of intrahepatic B16 melanoma and CT26 colon cancer cells without steatohepatitis. In the same experimental setup with additional diet-induced steatohepatitis, however, immunotherapy led to progressive tumor growth and a loss of CD4 T cells from the liver. The application of reactive oxygen species (ROS)-reducing N-acetylcysteine rescued the amount of intratumor CD4 T cells in mice with steatohepatitis and recovered therapeutic efficacy [108]. These results suggest an in situ mechanism of NASH with regard to failure on immunotherapies and furthermore identifies a putative strategy to overcome detrimental effects of NASH on CD4 T cell tumor immunity by protecting these cells from ROS-mediated damage. It will be intriguing to see whether the application of N-acetylcysteine is sufficient to restore tumor immunity in NASH-HCC patients and if this may even prevent NASH patients from CD8 T cell mediated liver damage and subsequent tumor development by reintroducing proper CD4-mediated regulation of CD8 T cell responses [107,108].

**Figure 2:**
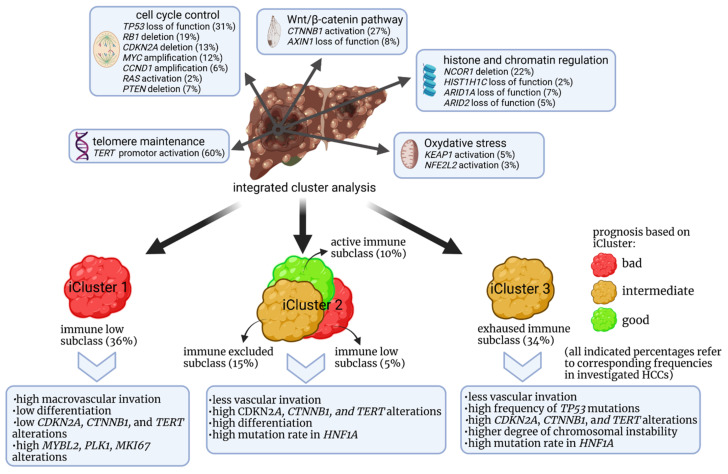
Proposed immune-genetic classification after integrated cluster analysis of HCC and influence on the prognosis established by the Cancer Genome Atlas Research Network [109].

In general, a broad genetic analysis of HCC samples could establish a correlative link between genetic features of the tumor and the prognosis. Such a study was performed by the research network of The Cancer Genome Atlas (TCGA) in a comprehensive manner by integrative genomic characterization of HCC [109]. The analysis of 196 HCCs revealed significantly mutated genes, such as *LZTR1*, *EEF1A1*, *SF3B1*, and *SMARCA4*. 22% of samples showed a high to moderate immune cell infiltration. However, overall survival was not significantly related to immune clustering. Alterations due to mutations or hypermethylation of genes that result in downregulation induced a metabolic reprogramming and, most importantly, the authors defined a genetic cluster that was associated with a poor prognosis. More data are required, however, to further support statistical significance. Figure 2 shows an overview of genetic HCC clusters that may have an influence on the prognosis. Treatment with CPI in combination with inhibition of angiogenesis suggests better outcomes for the exhausted/excluded subclass and the active immune subclass [109].

In summary, these studies suggest a tumor-specific T cell pool in HCC patients that is strongly attenuated by the tumor tissue and there appears to be a rather complex link to the genetic properties of HCC that affects T cell immunity and prognosis. Due to the plasticity of these T cells, or at least subpopulations of it, cytotoxicity can often be re-established by prudent selection of therapeutic means. Furthermore, if these cells could be expanded in vivo or ex vivo and subsequently directed to the tumor, this could provide a promising basis for T cell-based tumor therapy of HCC.

## 6. Other Immunotherapeutic Approaches of HCC

The liver being an exceptional organ when it comes to tolerance induction, this organ is mediating the ‘liver tolerance effect’ with regard to local and systemic tolerance to self and foreign antigens [110]. Liver cancer may exploit multiple mechanisms of this effect to ward off or silence tumor immunity. As already mentioned, senescence surveillance limits the outgrowth of pre-malignant hepatocytes [88]. However, if senescent cells are not cleared, they may give rise to HCCs that block maturation of CCR2^+^ myeloid cells. This cell type is required to execute the senescence program, and ablation of CCR2 leads to development of HCC. Inhibiting the maturation of myeloid precursors leads in turn to inhibition of NK cell functions and exacerbates HCC progression. Hence, the secretory phenotype of senescent hepatocytes leads to suppression of liver cancer in early stages of tumor development, but they may accelerate tumor progression in the late stages of HCC. It appears promising to investigate immunotherapies combining multiple strategies that include blocking the CCL2/CCR2 axis thereby enhancing NK cell infiltration and activity [111]. Loco-regional treatments in HCC are known to stimulate tumor immunity due to massive release of antigens from dying tumor cells. This may synergize with CPI and other immunotherapies. One study sought to trigger CD8 T cell immunity by ablative methods and used CPI to further stimulate T cell immunity. Ablation was performed by a TACE or radiofrequency ablation (RFA) combined with tremelimumab, a CTLA-4 inhibitor. The authors established this approach as a putative new treatment approach that leads to the accumulation of CD8 T cells with a correlation of a positive clinical activity [112]. Similarly, the combination of RFA with a dendritic cell vaccine based on monocyte-derived DCs stimulated with OK432 was well tolerated. This treatment combination improved TAA-specific T cell responses and the 5-year recurrence-free survival was significantly higher with 50% in the combined treatment group compared to 7.7% in patients without combined treatment [113]. Other clinical studies for HCC, e.g., IMMUTACE (TACE combined with nivolumab, NCT03572582), IMMULAB (RFA combined with pembrolizumab, NCT03753659), or IMMUWIN (selective internal radiation therapy (SIRT) combined with durvalumab (antibody specific for PD-L1), NCT04522544) are currently active to fathom loco-regional approaches with CPI. These and other studies (reviewed in [114]) will reveal synergies between established clinical treatment options with immunotherapies to improve the outcome for HCC patients.

Oncolytic virotherapy (OV) is a promising approach for the treatment of solid tumors. Viral vectors can be genetically modified to replicate in primarily in tumor tissue [115]. In pre-clinical models OV has shown promising results in combination with checkpoint inhibitors [116]. Mechanistically, OV appears to broaden the spectrum of tumor-directed T cell responses when combined with CPI. Viral replication in tumors induces expression of PD-1 on metastasis and inhibits dissemination, if mice were treated with PD-1 blocking antibodies in a liver cancer model [117]. In clinical settings, the oncolytic vector talimogene laherparepvec (T-vec), a herpes simplex virus type-1 armed with an expression cassette of granulocyte macrophage colony-stimulating factor (GM-CSF) to enhance antitumor immunity, has been used to treat patients with advanced melanoma in a phase III study [118]. This clinical study published in 2015 was the first phase III study with OV that led to approval of the FDA. Clinical studies investigating OV for the treatment of liver cancer have also been performed. The oncolytic and immunotherapeutic virus JX-549 (Pexastimogene devacirepvec or Pexa-Vec) based on a vaccinia virus also expresses GM-CSF and was evaluated in a randomized phase I/II dose-finding study. Pexa-Vec was well tolerated and showed tumor responses and dose-related survival in individuals with HCC [119]. In a subsequent phase IIb study, Pexa-Vec did not improve the overall survival of HCC patients as a second line treatment after a sorafenib failure. It was furthermore postulated that virotherapy has more potential in earlier disease stages [120]. At that time, pre-clinical studies appear particularly incongruent in comparison to clinical studies with regard to therapeutic efficiency of oncolytic virotherapy. However, first clinical studies of OV and CPI have been already performed in melanoma, in part with promising outcomes [121,122] and now there is also a combinatorial first line phase I/IIa study of oncolytic virotherapy (Pexa-Vec) with nivolumab in HCC patients ongoing (NCT03071094). Also other tumor entities such as glioblastoma show promising results for safety and efficacy in recent clinical trials with OV [123]. In light of these results and the probable high potency of OV especially in combination with CPI, new clinical trials should be encouraged to further improve the prognosis and therapeutic options for HCC.

Adoptive transfer of autologous lymphocytes derived from tumor tissue against overexpressed self-derived differentiation antigens has shown promising results in a subgroup of melanoma patients almost two decades ago [124]. The transferred cells were proliferating in vivo after ex vivo expansion, displayed functional activity, and were able to traffic to tumors. This proof-of-concept study invigorated a new therapeutic field of adoptive cell therapy (ACT). Since then, ACT of chimeric antigen receptor- (CAR-) T cells such as lisocabtagene maraleucel for refractory B cell lymphoma induced durable responses and a manageable long-term safety profile [125,126]. However, CAR T-cells can mediate severe adverse effects. Treated patients must be monitored closely for cytokine release syndrome and immune effector cell-associated neurotoxicity syndrome [127]. ACT comprises cells that mediates cellular immunity, such as CD8 T cells, iNKT cells (invariant NK T cells), γδ T cells, cytokine-induced immune killer cells, and CAR-T cells. Several clinical studies with ACT are being conducted. For example, a phase I/II study uses iNKT cells and PD-1^+^ CD8 T cells, that are assumed to be tumor specific, are used to treat various cancers, including HCC (NCT03093688). Other clinical studies use highly purified CTLs (cytotoxic lymphocytes) in combination with RFA (NCT02678013) or resection (NCT02709070) that have already reached primary completion. With regard to ACT of CAR-T cells, pre-clinical studies of patient-derived xenografts or orthotopic liver cancer, ACT of anti-GPC3 CAR-T cells have delivered positive results [128,129]. There are clinical studies ongoing (NCT04121273, NCT02905188, NCT03198546) that use GPC3 CAR-T cells. It has been shown that >70% of HCCs are positive for GPC3 and GPC3 expression is correlated with a poor prognosis [130]. Shi et al. published results from a first phase I CAR-GPC3 T cell study in 13 patients and found early signs of anti-tumor activity of these cells in HCC. The described safety profile included 9 patients with cytokine release syndrome [131]. One phase I study, that is applicable to HLA-A2^+^ patients, utilizes autologous genetically modified AFP^c332^T cells for the treatment of HCC (NCT03132792). First promising results have already been presented (overview for this and other ACT/HCC studies in [132]). In this clinical study, targeting AFP^+^ HCC tumors with AFP-specific CAR-T cells resulted in one complete response out of four patients and one patient had a partial response with 100% reduction of targeted tumors and only one non-targeted tumor nodule remained at therapy week eight. The application of CAR-T cells targeting a single antigen is likely to underlie immune escape and thus leading to treatment failure, especially when non-essential antigens for tumor survival are selected [46,133]. Hence, selection of multiple targets may lead to a higher success rate. For instance, study NCT03638206 impeded this putative pitfall and selected DR-5, C-met, and EGFR V III as CAR-T cell targets for the treatment of HCC. A comprehensive review including a list of clinical studies for HCC can be found elsewhere [134].

As one of the first vaccination approaches in HCC therapy, peptide immunizations have been employed to generate de novo cancer-specific T cell responses. Initial vaccination studies primarily focused on AFP, an oncofetal target which is expressed in approximately 50% of all HCCs. While the initial studies with AFP peptide-pulsed dendritic cells showed limited therapeutic efficacy [135], a more recent trial with AFP peptides emulsified in incomplete Freund’s adjuvant demonstrated clinical efficacy with one complete response and several patients with long-term disease control without severe side effects [136]. Since increased telomerase expression due to telomerase promoter mutations is a hallmark of HCC, vaccines targeting the catalytic telomerase subunit hTERT have been employed in a number of clinical trials. As an example, the peptide vaccine GV1001 targeting the hTERT epitope 611-626 was tested in a phase 2 trial in combination with GM-CSF and cyclophosphamide [137]. While the vaccinations were well-tolerated, no clear telomerase-specific T cells were detected, and clinical responses were limited. In another phase I study, HLA-A24 specific hTERT-specific peptides were used for adjuvant HCC treatment following radiofrequency ablation [138]. Side effects were mostly transient and limited to the skin while a trend towards lower cancer recurrence was noted in patients with detectable hTERT-specific immune responses. As a third prominent target, GPC3 has been subject of both preclinical and clinical trials due to its convincing specificity for HCC and its role as a negative prognostic factor. In an early phase I trial, intradermal peptide injection induced a partial response in one patient and a correlation between GPC3-specific immune responses and overall survival was noted [139]. Similar results were obtained in another phase I study in patients with advanced HCC with one partial response and several patients reaching stable disease [140]. These clinical trials highlight the potential of vaccination studies in HCC but reveal yet unsolved limitations regarding the quantity and quality of cancer-specific T cells induced by current vaccination regimens.

## 7. Prospects and Challenges for T Cell-Based Therapies

The review of current literature thus far may lead to deduction of five basic requirements that can be imagined for successful T cell-based therapies for HCC.

The first requirement is the identification and isolation of tumor-specific T cells. The source of these cells for the isolation process is usually tumor tissue or peripheral blood. Both sources face different challenges. Tumor tissue is a restricted source and immune low or immune excluded HCC may yield insufficient numbers of T lymphocytes. On the other hand, tumor tissue can also be a suitable source rich in tumor-specific T cells, depending on the entity and immunological landscape. Peripheral blood challenges the HLA-restricted identification process of tumor-specific T cells against TAA and neoantigens. The often much lower frequency in blood compared to tumor tissue is mitigated by the readily and abundant availability. Additionally, lymphocytes from blood are likely to be less impacted by the TME and consequently may preserve effector functions.

Secondly, T cells need to be expanded in vivo or ex vivo. Three approaches can be followed to expand ideally polyclonal T cells for therapeutic purposes. Prior to the isolation process, inhibiting the PD-1 pathway combined with anti-angiogenesis or other appropriate pathways, such as the CTLA-4 pathway already demonstrated good chances to trigger T cell responses with long term effects on tumor control in vivo. Next, ex vivo expansion of T cells derived from tumor tissue as proof-of-concept has been established by Rosenberg and colleagues for cancer therapy, as discussed above. The ex vivo approach also comprises identification and expansion of tumor-directed T cells from the periphery, as well as construction of CAR-T cells. In CAR-T cell regimens, selection of suitable tumor-targets is a crucial step to success. Another approach is vaccination as a third principle to induce and expand tumor-specific T cells.

The third requirement is trafficking and homing of adoptively transferred cells to the tumor. Few studies in rodents on how homing of expanded cells and the route of ACT applications affects outcome in liver cancer are available [141,142]. Homing of T cells on a molecular level requires correct interplay of cytokines from tumor tissue with cytokine receptors on T cells, rolling on the endothelium, and efficient extravasation and subsequent adhesion to the extracellular matrix [143]. Homing implies also proper engraftment within the host. ACT regimens often include lympho-depletion regimens prior to the cell transfer that influence the outcome. Hence, the route of application, lympho-depletion, and cellular features due to culture conditions and genetic manipulation needs to be optimized in regimens of ACT to reach optimal results.

Safety is the fourth basic requirement for T cell-based therapies and has utmost priority for the study design. Adverse events do occur in all regimens in which T cells are involved. Rosenberg reported autoimmunity in treated patients, such as vitiligo and uveitis. CPI may lead to numerous immune-related adverse events including autoimmunity that constitute a research field of its own [144]. Safety profiles of immunotherapies have been studied extensively allowing for precise treatment of severe adverse events that render these regimens manageable for most patients. (CAR-)ACT may account for cytokine release syndrome, tumor release syndrome, and, in case of CAR-modified T cells, for CAR-T cell related encephalopathy syndrome, cytopenia, infections, and immune effector cell-associated neurotoxicity syndrome [145].

**Figure 3:**
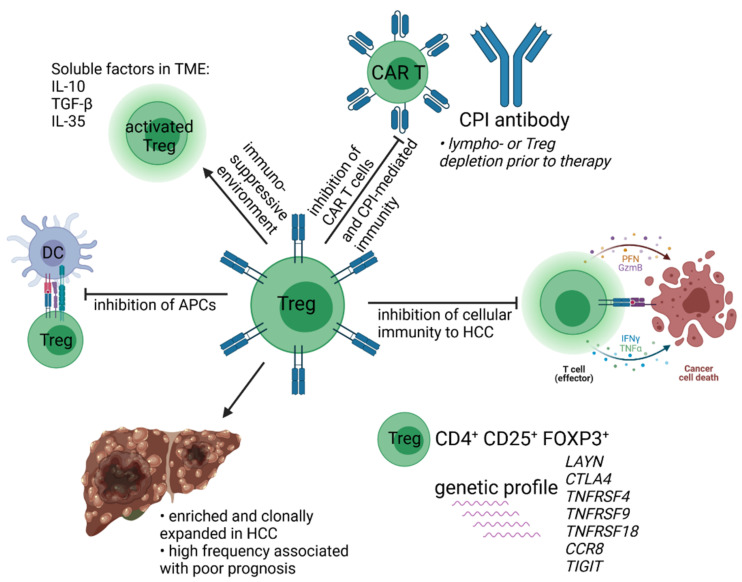
Aspects of Treg-functions in immunotherapies of HCC according to [55,79,145,147].

The fifth basic requirement is the management of overcoming tolerance mechanisms mediated by the TME that abrogate T cell immunity. Immunosuppressive cytokines and mediators such as IL-10, TGF-β, and adenosine may require inhibition or neutralization. Cellular components comprise MDSC, M2 macrophages, Tregs, and cancer-associated fibroblasts (CAFs) frequently produce other immunosuppressive mediators of T cell functions such as reactive oxygen/nitrogen species as well as arginase [146]. Tregs are of central importance for immunotherapies, as they are not only of prognostic value, but play a role in several aspects of therapeutic interventions that are addressed in Figure 3. Tumor-tissue chemo-attract Tregs that are expanded and differentiated locally and can mediate potent immune suppression in several tumor entities. This is detrimental to most immunotherapeutic approaches [147]. Hence, selective depletion may be required in these regimens and can be realized by application of low dose cyclophosphamide to allow for effective immunotherapy [148].

In general, strong inflammatory stimuli have been found to overcome effects of the TME. By using viral tumor infections, effective DC vaccination led to reduced levels of MDSC and significantly improved immunotherapeutic efficacies [149]. Additionally, other studies with OVs suggest a benefit for combinatorial approaches with immunotherapies [116,117,150]. The advantage of OV usage may be a ubiquitous inflammation and a concomitant release of dying tumor cells that dampens persisting tolerance mechanisms. This can provide a temporal window for induction of adaptive tumor immunity, thereby maintaining tumor inflammation that could keep immunosuppression further in check. Due to the heterogeneity of HCC, other approaches to overcome tolerance mechanisms would require analysis of suppressive pathways on an individual basis for a personalized therapy. Table 2 summarizes the issues discussed above and provides an overview of prospects and challenges of T cell-based therapies.

## 8. Conclusions

The shift in HCC treatment towards immunotherapy demonstrates the potential of immunity for therapeutic purposes. T cell-based therapies show promising results in subgroups of HCC patients. Biomarkers, however, that have been shown to be useful in other tumor entities fail to cover predictions in HCC. Improvement of current regimens for HCC need to be deduced from features of immunological landscapes and also from the environment of tumors embedded into cirrhosis or NASH. Different etiologies and the heterogeneous nature of HCC still need to be investigated to reveal novel immunotherapeutic targets and individualized approaches. Recent technological progress including single cell sequencing will continue to provide relevant information to realize these aims. The prospects and challenges of T cell-based therapies will surely teach important lessons in the field of immuno-oncology to ameliorate the outcome. An in-depth characterization of the complex network and interactions within the trinity of immune landscape, genetic features, and etiology will allow for identification of biomarkers that will guide appropriate treatment schemes with an improved prognosis for patients with HCC.

## Figures and Tables

**Table 1:** Overview of T cell phenotypes in HCC studies.

T Cell Phenotype	T Cell Function	Prognosis in HCC Patients	Study
FOXP3^+^ CD45^+^ including other lineages	Suppress CD8-mediated immunity; expression of TGF-β, VEGF, and IL-10	poor	[55]
PD-1^hi^ CD4 Treg and PD-1^hi^ CD8 Trm	More suppressive and exhausted in HBV-related HCC, reversible by CPI	poor for Treg better for Trm	[63]
tumor-infiltrating CD8 T cell	immune defence against tumor progression	good	[54,64]
CD8 T cell with high expression of inhibitory receptors: PD-1, TIGIT, TIM-3, LAG-3	exhaustion phenotype (Tex)	poor (for fully exhausted T cells)	[66,67]
PD-1^int^ TCF-1^+^ Eomes^lo^ CD8 T cell	early Tex, proliferative capacity, responsive to CPI	good under CPI treatment	[71,72]
PD-1^hi^ TCF-1^−^ Tbet^lo^ Eomes^hi^ CD8 T cell	terminally exhausted Tex, non-responsive to CPI	poor	[71,72]
CD8 T cell cluster *SLC4A10*	MAIT cells	poor, if frequency is low in HCC tissue	[79]
CD8 T cell cluster *CX3CR1*, *FCGR3A*, *FGFBP2*	effector T cells	n/d	[79]
CD8 T cell cluster *CTLA4*, *PDCD1*, *HAVCR2*	terminally exhausted Tex	n/d	[79]
CD8 T cell cluster *CTLA4*, *PDCD1*, *HAVCR2*, *GZMK*	Early Tex with putative cytotoxicity	n/d	[79]
CD4 Treg cluster *FOXP3*, *CTLA4*, *TNFRSF18*, *TNFRSF4*, and *CCR8*	T reg	no correlation found in this study	[79]
Tex and Treg cluster *LAYN*	suppressive function	poor when *LAYN* expression is high	[79]

**Table 2:** This table summarizes the prospects and challenges of T cell-based therapies.

	Prospects	Challenges
Identification and isolation of tu-specific T cells: TAAs and neoantigens	TAAs well defined for most HLA types neoantigens drive tumor responses, high frequency of tu-specific T cells	low frequency of TAA-specific T cells, monoclonal patient specific, time consuming id process, not clinically applicable yet, monoclonal
T cell expansion: in vivo and ex vivo	in vivo by CPI yields polyclonal responses, no id process necessary, rapid induction of immunity, agents off-the-shelf ex vivo from blood, well accessible source, putatively less influenced by the TME ex vivo from tumor-tissue, tumor-spec. T cells enriched	frequent non-response, temporal response, adverse events ex vivo from blood, requires id or modification by CARs, low initial frequency, monoclonal, adverse events ex vivo from tumor-tissue, time consuming culture/selection, exhausted T cells, limited availability, still experimental, adverse events
T cell homing	local administration: effective in targeted tumors systemic administration: simple route of application	less effective in non-targeted tumor nodules systemic transfer requires proper homing, putatively less effective than direct targeting in CAR-T cell therapies
safety	monitoring of adverse events well established majority of adverse events manageable	adverse events display high diversity of autoimmune disorders adverse events can pose life threatening complications in some patients
TME management: targeted intervention oncolytic virotherapy	complementing tumor immune cycle, expansion of response time, facilitates immunotherapies broadening spectrum of T cell responses, virus inflammation dampens tolerance induction of tumors, facilitates and promotes immunotherapies	precise intervention or characterization of tumor attributes required accessibility of tumors for OV injection required

## Data Availability

Not applicable.

## Abbreviations

ACT	adoptive cell transfer
AFP	alpha-fetoprotein
APC	antigen-presenting cell
CAF	cancer-associated fibroblast
CAR	chimeric antigen receptor
CD	cluster of differentiation
CI	confidence interval
CPI	checkpoint inhibition
CTL	cytotoxic lymphocyte
CTLA-4	cytotoxic T lymphocyte antigen 4
DC	dendritic cell
FDA	United States Food and Drug Administration
FOXP3	forkhead box protein P3
GM-CSF	granulocyte macrophage colony-stimulating factor
GPC3	glypican-3
HCC	hepatocellular carcinoma
HIF-1	hypoxia-inducible factor 1
HLA	human leukocyte antigen
hTERT	human telomerase reverse transcriptase
ICAM	intercellular adhesion molecule
IFN-γ	interferon gamma
IHC	immunohistochemistry
IL	interleukin
iNKT	invariant NK T cell
LAG-3	lymphocyte-activation gene 3
LAMP	lysosomal associated membrane protein
MAIT cell	mucosal-associated invariant T cell
MDSC	myeloid-derived suppressor cell
MHC	major histocompatibility complex
NAFLD	non-alcoholic fatty liver disease
NASH	non-alcoholic steatohepatitis
NY-ESO-1	New York-esophageal squamous cell carcinoma-1
ORR	objective response rate
OV	oncolytic virotherapy
PBMC	peripheral blood mononuclear cell
PD-1	programmed cell death antigen 1
PD-L1	ligand for programmed cell death antigen 1
Pexa-Vec	pexastimogene devacirepvec
RECIST	Response Evaluation Criteria in Solid Tumors
RFA	radiofrequency ablation
ROS	reactive oxygen species
SART	squamous cell carcinoma antigen recognized by T cells
SIRT	selective internal radiation therapy
TAA	tumor-associated antigen
TACE	transarterial chemoembolization
TAM	tumor-associated macrophage
TCGA	The Cancer Genome Atlas
TCR	T cell receptor
Teff	effector T cell
Tex	exhausted CD8 T cells
TGF-β	tumor growth factor beta
TIGIT	T cell immunoreceptor with Ig and ITIM domains
TIM-3	T cell immunoglobulin and mucin domain containing protein 3
TKI	tyrosine kinase inhibitor
TMB	tumor mutational burden
TME	tumor microenvironment
Tmem	memory T cell
Tregs	regulatory T cells
T-vec	talimogene laherparepvec
VCAM	vascular cell adhesion molecule
VEGF	vascular endothelial growth factor
VEGFR	vascular endothelial growth factor receptor.
